# The MacKinnon Lists Technique: An efficient new method for rapidly assessing biodiversity and species abundance ranks in the marine environment

**DOI:** 10.1371/journal.pone.0231820

**Published:** 2020-04-22

**Authors:** Lydia Luise Bach, David M. Bailey, Euan S. Harvey, Ross MacLeod

**Affiliations:** 1 Institute of Biodiversity, Animal Health and Comparative Medicine, College of Medical, Veterinary and Life Sciences, University of Glasgow, Glasgow, United Kingdom; 2 Department of Environment and Agriculture, School of Science, Curtin University, Perth, Western Australia, Australia; 3 School of Biological & Environmental Sciences, Liverpool John Moores University, Liverpool, United Kingdom; University of Waikato, NEW ZEALAND

## Abstract

Widespread and ever-increasing anthropogenic impacts in the marine environment are driving a need to develop more efficient survey methods for monitoring changes in marine biodiversity. There is a particular urgent need for survey methods that could more rapidly and effectively detect change in species richness, abundance and community composition. Here, test the suitability of the Mackinnon Lists Technique for use in the marine environment by testing its effectiveness for rapid assessment of fish communities. The MacKinnon Lists Technique is a time-efficient and cost-effective sampling method developed for studying avian tropical biodiversity, in which several list samples of species can be collected from a single survey. Using the well-established MaxN approach on data from deployments of a Baited Remote Underwater Video Systems for comparison, we tested the suitability of the MacKinnon Lists Technique for use in marine environments by analysing tropical reef fish communities. Using both methods for each data set, differences in community composition between depths and levels of protection were assessed. Both methods were comparable for diversity and evenness indices with similar ranks for species. Multivariate analysis showed that the MacKinnon Lists Technique and MaxN detected similar differences in community composition at different depths and protection status. However, the MacKinnon Lists Technique detected significant differences between factors when fewer videos (representing reduced survey effort) were used. We conclude that the MacKinnon Lists Technique is at least as effective as the widely used MaxN method for detecting differences between communities in the marine environment and suggest can do so with lower survey effort. The MacKinnon Lists Technique has the potential to be widely used as an effective new tool for rapid conservation monitoring in marine ecosystems.

## Introduction

Monitoring the abundance, diversity and distribution of species helps track the impacts of environmental disturbance, detect changes in population dynamics and enables effective management [1–3]. This requires accurate and precise information on species richness, abundance and assemblage composition, permitting the detection of community responses that might be caused by environmental change [4]. Such data also contributes to understanding the factors shaping community assemblages which can assist managers to make informed decisions [5,6].

In the marine environment a number of sophisticated methods such as mark and recapture, acoustic surveys or destructive methods have been developed to survey and monitor biodiversity for conservation and scientific purposes [7]. Many of these methods are costly and time intensive, requiring considerable expertise in terms of data collection and analysis [8–10]. Moreover, species assemblages in the marine environment are often characterised by high spatiotemporal variation and heterogeneity, making it difficult to fulfil the underlying assumptions of complex methodologies [9,11]. In many cases key conservation priority areas, such as coral reef environments, are characterised by high species richness and patchy distribution of key habitats and species. This adds considerable challenges to data collection, analysis and interpretation [9,12,13].

Marine environments, including temperate and coral reefs, are changing rapidly in response to climate change and other human disturbances [14,15], creating a need for methods which can rapidly assess these communities in a standardized and comparable manner [16]. A commonly used method for studying fish assemblages in coral reefs is the underwater visual census (UVC) conducted by divers. UVC has a range of limitations such as the divers’ impact on fish behaviour [17], effects of variation in diver swimming speed [18] and the need for trained divers that can immediately identify the species encountered and estimate their length [4, 19–21].

With the development of higher quality and relatively cheap video camera technology some of these limitations have been overcome, in particular the problems of consistent species identification [22–24]. With advances in computer power and software, the ability to carry out underwater photogrammetry, means that fish length and biomass estimates have greatly improved. Deployments of stationary video cameras are also used in conjunction with bait to attract fish to the camera [25–28].

One of the most common sampling approaches is to record the maximum number of individuals of each species seen at one time [29]. This value is known as the MaxN for that species and is considered an index of abundance. This approach was suggested by Cappo et al. (2003) and subsequently adopted by other teams in Australia and the US. The use of the MaxN approach avoids repeated counts of the same individual. However, because it only uses the maximum number of individuals at a single time it ignores much of the information recorded by the video [4]. Furthermore, the number of individuals detected at one time depends on behaviours of individual species. Changes in true abundance may not be detectable in species that only come to the bait in ones and twos and at higher densities fish may actively chase each other away [30]. Recognising that no survey method is without biases, it is useful to evaluate and compare methods of counting animals from terrestrial systems to see if these can be applied to marine systems. For example, the widely used Underwater Visual Census approach to sampling coral reef fish developed by Brock (1954) was a successful adaptation of visual counts of birds with an observer identifying and counting all the birds they saw along a transect [31].

Ideally, potential new sampling techniques should allow for analysis of both *in situ* data and video footage. They should also be comparable across survey methods, reduce the potential for double counting in UVC survey, use the data available in video footage to a greater extent, be widely applicable, fast and cost-efficient.

The MacKinnon Lists Technique (MLT) was developed for surveys of avifaunal communities in tropical forest ecosystems and has become an established technique for bird surveys, particularly in highly species rich communities [32–36]. The MLT can accumulate samples from any set of observational data where the order of individual detections can be recorded, and could therefore be used widely in the marine environment including for UVC surveys, baited and unbaited remote underwater video surveys.

We propose that MLT has unique features (further described below) that may make it useful in the marine environments, in particular in species rich habitats such as coral reefs. As such it is a highly flexible method to rapidly assess biodiversity *in situ* or using video, and, due to its simplicity, lower survey costs, staff time; availability of technology or training. Moreover, in comparison to MaxN more information is retained.

The MLT works by sequentially recording species detected during a survey in a standard-length list sample of unique species. To create a list sample, each species observed is recorded in order first seen until a pre-decided number of species is reached, normally either 5 or 10 unique species depending on the species richness of the study community [34,37]. A species can only be recorded once in each list sample. Once a list is completed, a new sample is begun, which can include species observed in the previous list(s). Typically, several lists are created during each survey effort (e.g. a transect or video recording), these lists are the sample units.

For birds, this technique has been shown to rapidly generate consistent species richness and relative abundance indices under a wide range of field conditions [34,37]. Bibby et al. (2000) argue that the MLT provides sampling units that are independent of collection time, observer expertise and spatial extent. This makes it a useful method to investigate changes in assemblage composition in space and time. Species relative abundance can be generated using MLT samples by calculating the proportion of samples each species occurs in. Previous studies suggest that the MLT is an efficient method to survey species groups of special interests such as species of conservation importance [37]. MacLeod et al. (2011) suggested that the MLT might be suitable for measuring differences in abundance and communities of many other taxonomic groups in addition to birds, including the marine environment.

In this study, we investigate for the first time the ability of MLT to rapidly generate monitoring data for marine fish communities, capable of 1) producing species richness and diversity estimates, 2) providing measures of relative abundance of species, including species targeted by fisheries, 3) detecting ecological relevant differences such as differences in community composition with depth and protection status and 4) its effectiveness at detecting changes in community composition as sampling effort decreases. In each case we compare MLT to results from the MaxN method, which is already widely used in marine science.

## Materials and methods

### Study area

Video footage for this study was collected in the Houtman Abrolhos Islands, located on the west coast of Western Australia, approximately 60 km offshore between 28°15’S and 29°S. The Houtman Abrolhos consists of four main island groups. This study took place in the Easter group, which lies South of North Island and the Wallabi Group but North of the Pelsaert group [4]. The Easter group study area includes an area (22.29 km^2^) closed to fishing which was established in 1994. For this study we used imagery collected between August and October 2005. Permits to conduct this work were obtained from the Department of Fisheries, Western Australia, who also provided logistical assistance.

#### Survey work

Imagery for this study was collected by baited remote stereo-video systems, filming for one hour. Video cameras were deployed in four sites, three of which were open to fishing and one was closed to fishing within the reef observation area (ROA). Within each of these at least five replicate deployments were made, which were split between shallow (8–12 m) and deep (22–26 m) reef slopes. Therefore, survey work resulted in 34 one-hour videos from a three-factor experimental design: protection status (St, two level fixed factor: fished or ROA), depth (De, two level fixed factor: deep (22–26 m) or shallow (8–12 m)) and site (S, nested random factor). This work was conducted by Warson et al. (2007). To account for correlation between lists within the same videos, we also added video as a random factor for MLT.

Survey sites were standardized with each site representing the same general habitat (predominantly coral) and deployments were made randomly within these sites. Each deployment site was separated by at least 250 m in order to minimize the chances of individual fish from moving between sites. Surveys were carried out between 0800 and 1600 hours.

### Image analysis

Each video was viewed in the video analysis program EventMeasure [38] and the following information extracted. For MaxN, each individual or group of individuals were identified to species level and then the maximum number of individuals of each species in the field of view at any one time was established for each video [26]. In line with other studies for MLT [32,34], we generated a chronologically ordered master list by recording a list of all individuals seen during a video. To simplify recording, species had to be out of field of view for more than three minutes before the same species was added as a new record. This avoided having to record long sequences of a species from a single individual passing repeatedly through the field of view. This was for convenience and is not an essential part of the technique, as repeated records of the same species would in any event be eliminated at the next stage of the sampling process. Once the data was assembled into this time ordered master list, we separated it into list samples consisting of five species each. A list sample size of five species was selected rather than ten species which is more common in avian studies, as the fish community species richness was less than found in most bird communities to which this method has been applied (most bird communities surveyed comprised between 150 and 300, compared to approximately 90 fish species associated prior work conducted in our sampling location) [34,37]. Each list sample provides a sample of the overall community present at a unique combination of time and space, as each sample is made up of a fixed number of species it represents a fixed proportion of the overall community studied. To ensure all data from the master list were used to estimate species richness for each habitat (i.e. the same status and depth category), partial list samples from individual videos (where less than five species were found at the end of a video) were pooled and added as additional lists for each habitat. Additional lists were not analysed as part of the multivariate analysis as video was being used as a random factor.

### Statistical analysis

#### Species richness estimation

Observed and estimated species richness accumulation curves for MaxN (per video sample for the factors status and depth) and MLT (per list sample for the factors status and depth) were generated using EstimateS v. 9.1 [39]. In order to remove sample order effects, average observed species richness (Sobs accumulation curve) was calculated by bootstrapping order species 50 times. Species richness estimators were then used to predict number of species within each habitat, with curves generated indicating if the area was sufficiently sampled. We selected ACE, ICE, Chao 1, Chao 2, Jack 1, Jack 2m MMruns and MMMeans species richness estimators as previous studies have suggested that these estimators produce the most consistent predictions over a range of species richness values [37].

#### Community diversity and evenness

Fisher’s alpha [40], Pilou’s J evenness [41], and Brillouin index for evenness [41] and diversity were calculated for MaxN (sample unit being video within a habitat) and MLT (sample unit being a list sample within a habitat) using the Diversity4 package. Standard deviations of the abundance indices were calculated using Diversity4. The equations used to calculate the indexes are based on published sources [42,43].

#### Relative abundance indices for common and target species

Comparisons between methods were made using the ten species with the highest relative abundance index for each method within each habitat. We also calculated the relative abundance within each habitat of species commonly targeted for fishing. MaxN and MLT species abundance indices were calculated as average MaxN and total abundance count for MLT (sum of all lists), per video in each of the four habitat types.

#### Multivariate analysis

Community assemblage data were analysed with permutational multivariate analysis of variance (PERMANOVA), in the PRIMER 6 statistical package [44]. Relative abundance based on MaxN and MLT were analysed separately according to a three—factor design (MaxN) and four—factor design (MLT), as described above. Prior to analysis this data was square root transformed and a dummy variable was added. The analysis used Bray Curtis distance dissimilarly. Permutational distance based approaches are of advantage when analysing abundance data as these tend to have many zero counts and are highly skewed [45,46]. This enabled the examination of significant factors influencing the abundance data. In order to understand the ability of each technique to discriminate patterns and distinguish between factors at lower sampling efforts, we analysed a lower number of videos within each habitat according to a balanced design with five, three and two videos per habitat. Videos were chosen randomly, but were the same for both methods. At these lower sampling efforts, we generated p-values for both methods using a Monte Carlo random samples from the asymptotic permutation distribution [47].

## Results

### Species richness and diversity measurement

**Fig 1:**
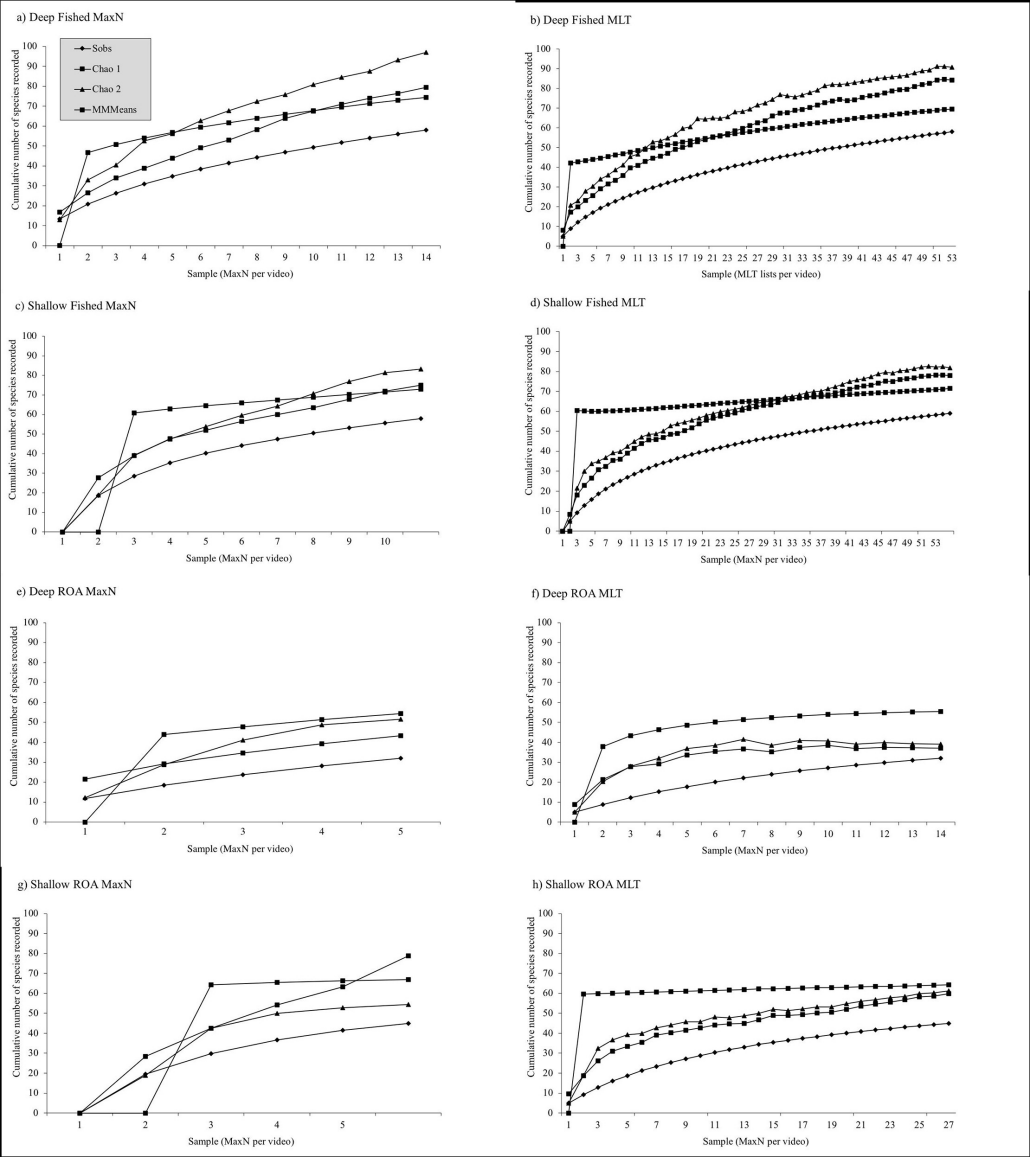
Species accumulation curves based on MaxN and MLT for four coral reef fish habitats.

The MLT consistently generated more samples across each of the habitats, with for example 53 list samples compared to 15 video samples in the Deep Fished habitat (Table 1). This is because the MLT makes use of more of the observations captured in each video allowing several list samples (each of which contains five species) to be complied from a single video. Using these samples both methods yielded similar estimated species richness in each habitat (Paired t-test: t = 0.80, df = 3, p = 0.48, Table 2). However, the greater number of MLT samples appeared to result in species richness estimates and species accumulation curves levelling off to a greater extent compared to MaxN thus providing more stable estimates of community species richness in each habitat (Table 1 and Fig 1). This was investigated further using the sample-based Chao2 species richness estimator, as this enables confidence interval calculation for species richness estimates. In the Deep Fished, Shallow Fished and Deep ROA habitats, the MLT Chao2 species richness estimate appeared to have stabilised by the final samples with the last three, five and three samples respectively providing species richness estimates that differed by less than one species (Table 1, S1 Table). For Shallow ROA the MLT Chao2 species richness estimate was still changing by slightly more than one species per sample in the final samples suggesting more sampling would be needed to produce a stable species richness estimate. In all four habitats Chao2 species richness estimate was still changing between the final two samples for MaxN, with a change between estimates of four species for Deep Fished, two species for Shallow Fished, three species for Deep ROA and two species for Shallow ROA (Table 1 and supplementary materials). Even with only four habitat comparisons available this difference in the final rate at which species richness estimates were changing was very close to significant between the two methods (Paired t-test: t = 3.0, df = 3, p = 0.058), providing evidence of an underlying difference in efficiency of methods. For the MLT Chao 2 species richness estimates the range of the 95% confidence intervals was also somewhat smaller than for MaxN for three out of the four habitats (95% CI Range: Deep Fished MLT 76.7 v MaxN 88.8, Shallow Fished MLT 58.4 v MaxN 62.1, Deep ROA MLT 23.2 v MaxN 54.3, Shallow Fished MLT 47.2 v MaxN 26.8).

**Table 1:** Samples generated by MaxN and MLT per habitat and stability of species richness (SR) estimates. As described in the methods, based on the master list, partial list samples at the end of videos were added to form additional pooled list samples for a habitat. Total number of additional lists generated is given in brackets.

** **	**Number of video samples generated**	**MaxN Final SR Estimate Chao 2**	**MaxN Penultimate SR Estimate**	**MaxN Final Rate of SR Change**
Deep Fished	14	97.00	93.18	3.82
Shallow Fished	10	83.30	81.52	1.78
Deep ROA	5	51.54	48.72	2.82
ShallowROA	5	54.42	52.76	1.66
** **	**Number of list samples generated (pooled lists in brackets)**	**MLT Final SR Estimate Chao 2**	**MLT Penultimate SR Estimate**	**MLT Final Rate of SR Change**
Deep Fished	53 (6)	90.70	91.13	0.43
Shallow Fished	54 (4)	81.90	82.44	0.54
Deep ROA	14 (1)	39.04	39.21	0.17
ShallowROA	27 (2)	61.37	60.23	1.14

**Table 2:** Species richness estimates for each habitat. Based on species estimators (S(exp), ACE, ICE, Chao1, Chao2, Jack1, Jack2 and MMruns).

Habitat	Deep fished		Shallow fished		Deep ROA		Shallow ROA	
Index	MaxN	MLT	MaxN	MLT	MaxN	MLT	MaxN	MLT
**S(exp)**	58.00	58.00	58.00	59.00	32.00	32.00	45.00	45.00
**ACE**	79.00	82.16	74.4	81.21	50.27	45.15	51.84	59.36
**ICE**	105.35	92.45	83.56	79.08	66.68	49.38	62.35	61.6
**Chao 1**	79.34	84.19	74.97	77.97	43.30	37.04	78.92	59.96
**Chao 2**	97.00	90.70	83.30	81.90	51.54	39.04	54.42	61.37
**Jack 1**	84.00	82.53	78.70	79.61	47.20	45.00	59.40	61.37
**Jack 2**	101.67	99.03	91.41	92.27	55.90	48.30	64.80	70.88
**MMruns**	83.49	69.59	74.28	71.87	69.10	55.45	83.66	64.74

Fisher’s alpha (all sample index), Brillouin Diversity, Brillouin Evenness and PilousJ evenness were calculated for each habitat (Table 3). Based on the widely overlapping standard errors the values for both methods are very similar with both methods identifying the same pattern, with Deep Fished and Shallow Fished habitats characterised by greater species diversity, but similar evenness compared to those in the ROA.

**Table 3:** Diversity and evenness indices for MaxN and MLT. Fishers alpha index, Brillouin Diversity, Brillouin Evenness and PilousJ evenness for community diversity and evenness were obtained from Diversity 4 for both techniques including Jacknife Standard Error across the four habitats.

Habitat type	Fishers alpha (+- Jacknife SE)	Brillouin Diversity (+- Jacknife SE)	Brillouin Evenness (+- Jacknife SE)	PielouJ Evenness (+- Jacknife SE)
**Max N**				
Deep Fished	16.19 (2.45)	3.10 (0.16)	0.81 (0.03)	0.80 (0.04)
Shallow Fished	15.19 (1.77)	3.00 (0.15)	0.77 (0.06)	0.77 (0.04)
Deep ROA	12.42 (3.77)	2.19 (0.25)	0.70 (0.12)	0.69 (0.08)
Shallow ROA	12.50 (2.38)	2.16 (0.20)	0.60 (0.05)	0.60 (0.05)
**MLT**				
Deep Fished	17.35 (1.79)	3.13 (0.09)	0.82 (0.02)	0.81 (0.02)
Shallow Fished	15.83 (2.09)	2.96 (0.18)	0.76 (0.05)	0.76 (0.05)
Deep ROA	12.70 (1.87)	2.16 (0.38)	0.69 (0.16)	0.68 (0.13)
ShallowROA	12.74 (2.63)	2.18 (0.41)	0.61 (0.12)	0.61 (0.12)

### Abundant species and target species

We compared the ten most abundant species (numerically) for MLT and MaxN (Table 4). Both methods identified very similar lists of the most abundant ten species. For each habitat, the methods agreed on 9 out of 10 of the most abundant species and for Shallow ROA provided agreement on 10 out of 10. Species ranks within the lists were also very similar, with an average difference of one rank or less between the methods in each of Deep Fished, Shallow Fished, Deep ROA and Shallow ROA.

**Table 4:** Most abundant species in the four coral reef fish communities according to MaxN and MacKinnon Lists Technique. The rank of the top ten species is indicated in brackets.

	MaxN	MLT	MaxN	MLT	MaxN	MLT	MaxN	MLT
	Deep Fished	Deep Fished	Shallow Fished	Shallow Fished	Deep ROA	Deep ROA	ShallowROA	ShallowROA
*Chaetodon assarius*	23 (8)	20 (8)	0	0	0	0	0	0
*Chaetodon lunula*	0	0	0	0	3 (9)	4	11 (8)	6 (9)
*Chaetodon plebeius*	0	0	0	0	3 (10)	2 (9)	0	0
*Chlorurus sordidus*	0	0	69 (2)	58 (2)	5 (7)	5 (6)	13 (6)	13 (5)
*Choerodon rubescens*	39 (4)	30 (3)	18 (9)	16 (10)	7 (4)	6 (5)	12 (7)	12 (6)
*Chromis westaustralis*	23 (7)	23 (5)	137 (1)	134 (1)	64 (1)	63(1)	218 (1)	203 (1)
*Coris auricularis*	37 (5)	22 (6)	38 (5)	24(8)	0	0	0	0
*Dascyllus trimaculatus*	0	0	28 (8)	26 (7)	0	0	0	0
*Gymnothorax woodwardi*	0	0	8	15 (9)	4 (8)	4 (7)	0	0
*Kyphosus cornelii*	0	0	0	0	0	0	42 (2)	43 (2)
*Lethrinus nebulosus*	0	0	0	0	6 (6)	3 (8)	17 (4)	17 (4)
*Pagrus auratus*	67 (2)	26 (4)	0	0	7 (5)	7 (4)	0	0
*Parupeneus spilurus*	20 (9)	18	0	0	0	0	0	0
*Pentapodus nagasakiensis*	16	16 (10)	0	0	0	0	0	0
*Plectropomus leopardus*	46 (3)	42 (2)	30 (7)	30 (4)	12 (2)	11 (2)	9 (9)	12 (8)
*Pseudocaranx* spp	68 (1)	62 (1)	56 (3)	56 (3)	0	0	0	0
*Scarus ghobban*	20 (10)	18 (9)	0	0	0	0	0	0
*Scarus schlegeli*	27 (6)	21 (7)	33 (6)	26 (6)	0	0	7 (10)	6 (10)
*Scombridae spp*	0	0	0	0	9 (3)	8 (3)	19 (3)	19 (3)
*Stethojulis strigiventer*	0	0	0	0	2	2 (10)	0	0
*Thalassoma lunare*	0	0	42 (4)	29 (5)	0	0	16 (5)	10 (7)
*Thalassoma lutescens*	0	0	17 (10)	16	0	0	0	0

**Fig 2:**
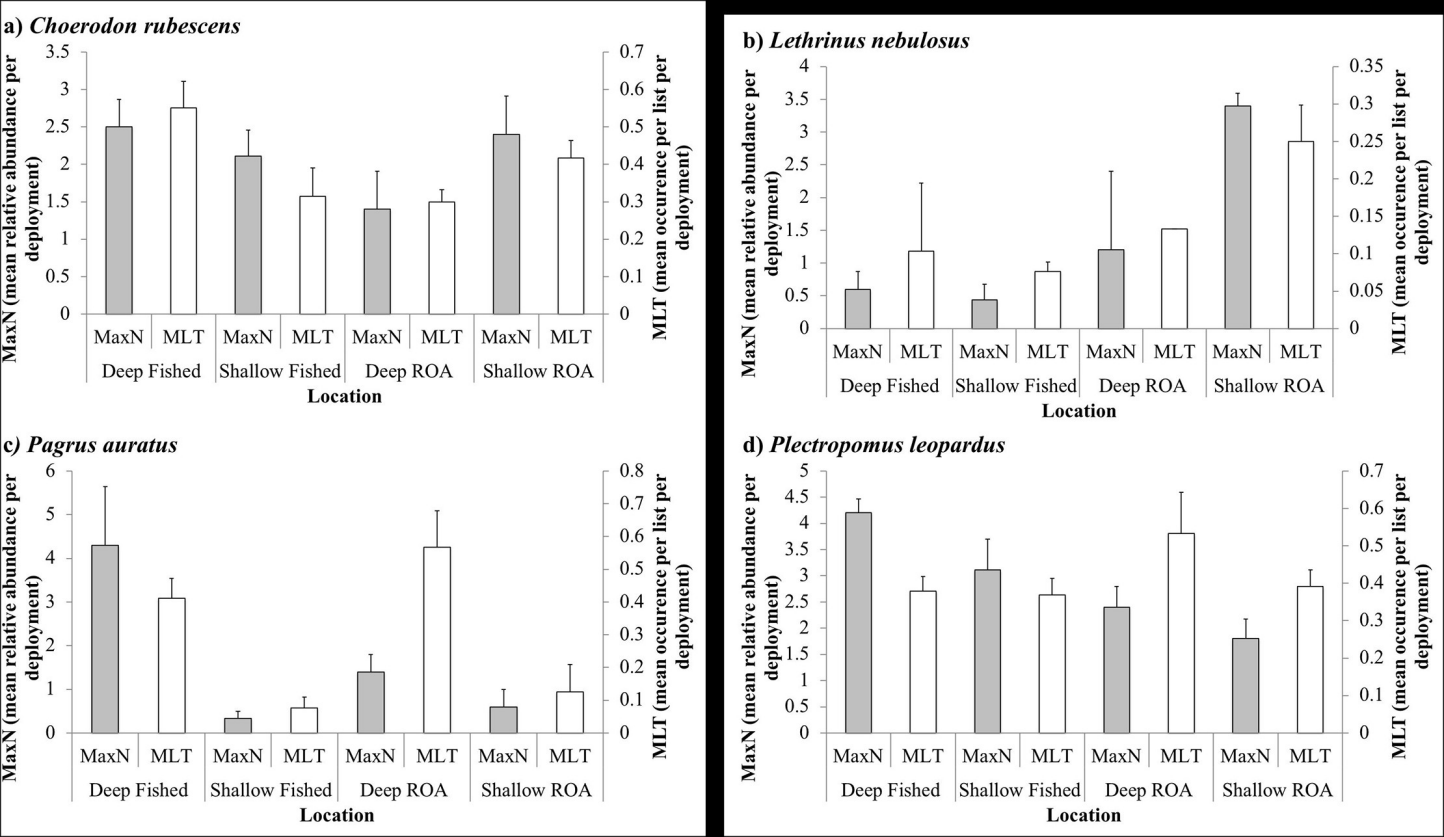
Mean relative abundance for MaxN (average MaxN per video deployment) and MLT (fraction of lists the species occurred in within videos) in each habitat of the most important fishing targeted species.

The mean relative abundance of four species targeted for fishing was calculated per habitat for both methods. Again, the methods identified very similar patterns of species abundance across different habitats (Fig 2).

### Multivariate analysis

The square-root transformed relative abundance data generated from all the deployments with each method analysed separately, showed the same significant differences in fish assemblage composition for the factors conservation status and depth with both methods. The random factor video was highly significant for MLT (Table 5).

**Table 5:** Comparison of ability of MaxN and MTL methods to detect significant effects on community composition. PERMANOVA results of square root transformed relative abundance data generated by MaxN and MLT using Bray Curtis dissimilarity matrix and one dummy variable. Significant values are highlighted bold.

**Source**	**Df**	**MS**	**Pseudo-F**	**P(perm)**
**MaxN**				
Status	1	6528.5	4.1	**0.007**
Depth	1	8623.2	5.3	**<0.001**
StatusxDepth	1	3424.4	2.1	0.051
Site(Status)	8	1507.9	0.8	0.810
DepthxSite(Status)**	7	1576.5	0.8	0.760
Residual	9	1902.8		
Total	27			
**MLT**				
**Source**	**Df**	**MS**	**Pseudo-F**	**P(perm)**
Status	1	7373.4	2.4	**0.007**
Depth	1	9207.4	3.2	**0.002**
StatusxDepth	1	4484.1	1.6	0.100
Site(Status)	8	3070.2	1.0	0.610
Video(Site(Status)xDepth)	17	3296.2	1.3	**0.006**
Res	98	2604.1		
Total	134			

Following this analysis, we randomly dropped the number of videos used in the analysis, allowing us to investigate how MaxN and MLT perform at lower sampling efforts (Table 6). Both techniques found significant differences between status and depth at a balanced sampling effort of five video deployments per habitat. However, MLT found a highly significant difference for the interaction between status and depth. MLT continued to detect the effect of protection status, depth and their interaction as significant with a further reduction in sampling effort to three videos per habitat. While MaxN only detected a significant effect of status with no significant differences between depth and no interactions.

**Table 6:** Comparison of ability of MaxN and MTL methods to detect significant effects on community composition with lower sampling effort. PERMANOVA results of square root transformed relative abundance data generated by MaxN and MLT. Significant values are highlighted in bold. The full experimental design was reduced to five videos for all habitats. By reducing the sample size of the fished sites at both depths to five, maintaining ROA samples at five, following by reducing fished and ROA video deployments to three and ultimately two. P(MC) denotes Monte Carlo permutations. Significant values are highlighted in bold.

Video/ habitat	MaxN						MLT				
**5**	**Source**	**df**	**MS**	**Ps-F**	**P(MC)**		**Source**	**df**	**MS**	**Ps-F**	**P(MC)**
	Status	1	5923.7	3.8	**0.014**		St	1	7841.9	2.3	**0.010**
	Depth	1	7087.4	4.1	**0.010**		De	1	8569.1	3.5	**0.001**
	Site(Status)	6	1537.6	0.8	0.711		Si(St)	5	2972.8	0.9	0.613
	StatusxDepth	1	3503.2	2.0	0.096		StxDe	1	4324.5	1.9	**0.028**
	DepthxSite(Status)	5	1738.1	0.9	0.593		DexSi(St)	5	2101.8	0.7	0.966
	Residuals	4	1902.7				Vi(Si(St)xDe)	7	3338.0	1.2	0.089
	Total	18					Res	61	2710.4		
							Total	81			
**3**	**Source**	**df**	**MS**	**Ps-F**	**P(MC)**		**Source**	**df**	**MS**	**Ps-F**	**P(MC)**
	Status	1	2932.3	2.5	0.086		St	1	4864.6	2.8	**0.005**
	Depth	1	5683.7	3.0	0.072		De	1	10760.0	8.2	**0.001**
	Site(Status)	3	1134.0	0.5	0.842		Si(St)	2	1480.2	0.4	0.991
	StatusxDepth	1	4273.6	2.2	0.121		StxDe	1	6526.5	5.9	**0.001**
	DepthxSite(Status)	3	1858.9	0.9	0.613		DexSi(St)	2	836.0	0.2	0.999
	Residuals	2	2185.7				Vi(Si(St)xDe)	4	3808.9	1.4	**0.049**
	Total	11					Res	41	2751.6		
							Total	52			
**2**	**Source**	**df**	**MS**	**Ps-F**	**P(MC)**		**Source**	**df**	**MS**	**Ps-F**	**P(MC)**
	Status	1	2976.4	2.7	0.202		St	1	3214.4	2.0	0.119
	Depth	1	5159.4	2.5	0.233		De	1	6017.1	3.6	**0.017**
	Site(Status)	1	1023.0	0.5	0.697		Si(St)	1	1713.1	0.4	0.923
	StatusxDepth	1	2349.7	1.4	0.413		StxDe	1	3365.5	2.5	0.053
	DepthxSite(Status)	1	1693.2	0.8	0.538		DexSi(St)	1	1400.3	0.4	0.961
	Residuals	2	2185.7				Vi(Si(St)xDe)	2	4375.9	1.5	0.065
	Total	7					Res	26	2833.6		
							Total	33			

## Discussion

For the first time, we have tested the ability of the MacKinnon Lists Technique to generate useful results on biodiversity patterns in marine fish communities. Our results show that this new approach is able to generate comparable results to the well-established MaxN methodology, with species richness estimates, diversity indices, relative abundance and assemblage composition results similar between the two methods. Moreover, MLT continued to detect more key variables as significant effects compared to the MaxN methodology as sampling effort was reduced. Due to the greater use of data available in video surveys, the MLT appeared to produce more stable estimations of species richness, suggesting that reliable assessments of biodiverse communities could be achieved with lower sampling effort.

These results suggest MLT is a viable method to assess spatial or temporal changes in species richness, relative abundance and community composition in marine environments and therefore could be a valuable tool for rapid conservation assessments in marine environments and possibly more widely under other circumstances where resources for sampling are limiting.

The consistency of both methods in generating similar ranks of the most abundant species and in generating comparable patterns of relative abundance for species of key conservation concern suggest that MLT should be a useful tool to assess the relative abundance of target species. This is encouraging not only for surveys in the marine environment, but also more generally, as previous tests on highly diverse tropical avian communities have often struggled to collect sufficient data from multiple methods to compare relative abundance ranks of more than a few species [35,37].

The choice of sampling technique and method of analysis for biodiversity assessments in general often depends on the researcher’s experience and preference, budget, study aim, focal species and a choice between different biases associated with different techniques [12]. Fjeldsa (1999) advocates the use of MLT for birds as being a highly time-efficient method as lists samples can be continuously generated while randomly moving through a habitat. This is a potentially significant advantage of the MLT compared to other methods traditionally used in avian studies, such as point counts where the time moving between survey points can significantly reduce data collection time [37].

In the context of field surveys whether in terrestrial or marine environments, MLT could allow a surveyor to cover a greater survey area in less time, generating a greater number of samples and often will require almost no prior preparation time for laying out survey grids or lines. In this study, the effort needed to analyse video footage to calculate relative abundance and species richness was similar for both methods (one-person hour per 60 min video). When measuring species richness and relative abundance, both methods require little technology and are comparable in terms of time required for analysis. Therefore, both methods are likely to be feasible options in environments where survey costs, staff time, availability of technology and training is limited. In a snorkelling and diving context, the MLT may allow for a faster and more standardized sampling approach, without the challenge of considering time restrictions, swimming speed or transect length, therefore making it a much simpler approach that is easier to implement in a standardised manner.

In a real-world context, areas of conservation importance often lack expertise and equipment to fully assess fish community composition. MLT has been shown to generate consistent relative abundance estimates across a range of personnel experience [34,37]. We suggest that using MLT in the marine environment could allow personnel with a lack experience or scientific support to focus on being able to confidently identify species of key conservation importance in the field, rather than on the more complex methodological requirements of other techniques. This should then enable such observers to help assess the spatial and temporal variation in fish assemblage composition more reliably, a key aim of many rapid assessment surveys and for conservation monitoring.

It is worth also noting that because it collects multiple samples per video the MLT technique may sample solitary fish species to a greater extent than MaxN, which only focuses on the maximum group size seen per video. This would make MLT a useful tool for assessing changes in relative abundance of solitary and numerically less common species, which would be consistent with data generated from terrestrial surveys [37]. In contrast, it is likely that the focus on maximum group size will mean the MaxN technique will more readily detect changes in relative abundance of fish species that frequently move in large groups. For this reason, we suggest that, where sufficient funds are available, an effective approach to marine biodiversity assessments might be to use both the MLT and MaxN methods together to analyse videos, diver or other surveys and report the results of both so that the strengths of each complement each other and make the most of the data available.

An important aspect of the MLT is that as a sampling with replacement methodology, it does not require all redetections of the same fish to be eliminated from the analysis. Most methods of assessing biodiversity patterns can be used with sampling with replacement methodologies that are not invalidated if some individuals are redetected. Here, we used a set of rules to reduce redetections (i.e. a species had to have been out of the field of view for > three minutes before the same species was added to a new list). Although a useful time-saving step during processing of the videos this is not essential to the method.

As with all methods, MLT has some limitations. As such, it should be taken in consideration that MLT tends to weight regularly spaced territorial species as more abundant than schooling species, which can affect the calculation of diversity indices and may result in the distribution of relative abundances to appear more even than using other methods such as MaxN (which is likely to estimate solitary species and species abundance and makes it challenging to quantify sampling area in particular when bait is used). Moreover, Pourson (1997) noted that while MLT is a useful tool to determine sampling effort and species richness, differences in species detectability mean that relative abundances can only be compared within species across habitats or sites. The importance of considering similar habitats when making comparisons has been noted by others previously [16,35,36].

There are currently a number of useful methods available to monitor and compare fish assemblage composition, including MaxN. The results of our study suggest that MLT is also likely to be a useful technique for the assessment of fish assemblages, enabling rapid assessment of spatial and temporal variation in species relative abundance, and one that may complement existing methods. The MLT method is a promising tool to collect biodiversity survey data or analyse video footage in aquatic environments where there is a limited budget, staff time, available technology and conditions might be too challenging to maintain some other types of standardized sampling approach. In particular, we suggest MLT could be considered for difficult to standardize conditions such as transects in coral reef and other marine applications such as diver and un-baited video or camera surveys.

In this study, as well as providing the first test of the MLT for marine sampling, we also carried out the most comprehensive comparison to date between MLT and an existing biodiversity sampling methodology. By showing that species richness estimates, diversity indices, relative abundance and assemblage composition results were all consistent across methods our results are likely to be useful not just in the marine context but also for biodiversity surveys in general. We therefore suggest that the MLT methodology is likely to be effective not just for coral reef fish, for bird communities and amphibian communities (49), but also in other species-rich communities where biodiversity needs to be sampled cheaply, quickly and efficiently for conservation monitoring or other purposes.

## Supporting information

S1 Table Chao 2 species richness estimative for all samples within each habitat and rate of change in richness estimate. (DOCX)

S1 Dataset Data set showing mean relative abundance per video deployment across status, site and depth (MaxN). (XLSX)

S2 Dataset Data set showing lists of species per video across status, site and depth (MLT). (XLSX)
