# Supplementary material for: The MacKinnon Lists Technique: An efficient new method for rapidly assessing biodiversity and species abundance ranks in the marine environment
Source: PLoS One. 2020 Apr 22;15(4):e0231820. doi: 10.1371/journal.pone.0231820 (PMC7176086; doi:10.1371/journal.pone.0231820)
Supplement: S1 Table — (DOCX) [file pone.0231820.s001.docx]

**S1 Table1. Chao 2 species richness estimative for all samples within each habitat and rate of change in richness estimate**.

| **Habitat** |  | **MaxN** | | **MLT** | |
| --- | --- | --- | --- | --- | --- |
|  | **Sample number** | **Chao 2 Mean** | **Rate of Change of Species Richness Estimate** | **Chao 2 Mean** | **Rate of Change of Species Richness Estimate** |
| **Deep Fished** | 1 | 12.99 |  | 4.97 |  |
|  | 2 | 32.90 | 19.91 | 20.67 | 15.70 |
|  | 3 | 40.55 | 7.65 | 22.92 | 2.25 |
|  | 4 | 52.48 | 11.93 | 27.96 | 5.04 |
|  | 5 | 56.23 | 3.75 | 30.26 | 2.30 |
|  | 6 | 62.58 | 6.35 | 34.05 | 3.79 |
|  | 7 | 67.74 | 5.16 | 36.08 | 2.03 |
|  | 8 | 72.40 | 4.66 | 38.73 | 2.65 |
|  | 9 | 75.76 | 3.36 | 41.17 | 2.44 |
|  | 10 | 80.90 | 5.14 | 45.48 | 4.31 |
|  | 11 | 84.51 | 3.61 | 46.57 | 1.09 |
|  | 12 | 87.57 | 3.06 | 49.61 | 3.04 |
|  | 13 | 93.18 | 5.61 | 52.73 | 3.12 |
|  | 14 | 97.00 | 3.82 | 53.38 | 0.65 |
|  | 15 |  |  | 54.79 | 1.41 |
|  | 16 |  |  | 56.63 | 1.84 |
|  | 17 |  |  | 59.71 | 3.08 |
|  | 18 |  |  | 60.61 | 0.90 |
|  | 19 |  |  | 64.50 | 3.89 |
|  | 20 |  |  | 64.39 | -0.11 |
|  | 21 |  |  | 65.00 | 0.61 |
|  | 22 |  |  | 64.90 | -0.10 |
|  | 23 |  |  | 65.71 | 0.81 |
|  | 24 |  |  | 68.03 | 2.32 |
|  | 25 |  |  | 68.24 | 0.21 |
|  | 26 |  |  | 69.54 | 1.30 |
|  | 27 |  |  | 71.45 | 1.91 |
|  | 28 |  |  | 72.59 | 1.14 |
|  | 29 |  |  | 74.47 | 1.88 |
|  | 30 |  |  | 76.79 | 2.32 |
|  | 31 |  |  | 76.20 | -0.59 |
|  | 32 |  |  | 75.71 | -0.49 |
|  | 33 |  |  | 76.59 | 0.88 |
|  | 34 |  |  | 77.80 | 1.21 |
|  | 35 |  |  | 79.11 | 1.31 |
|  | 36 |  |  | 81.35 | 2.24 |
|  | 37 |  |  | 81.91 | 0.56 |
|  | 38 |  |  | 82.02 | 0.11 |
|  | 39 |  |  | 82.35 | 0.33 |
|  | 40 |  |  | 82.93 | 0.58 |
|  | 41 |  |  | 83.67 | 0.74 |
|  | 42 |  |  | 84.14 | 0.47 |
|  | 43 |  |  | 85.10 | 0.96 |
|  | 44 |  |  | 85.38 | 0.28 |
|  | 45 |  |  | 85.89 | 0.51 |
|  | 46 |  |  | 86.23 | 0.34 |
|  | 47 |  |  | 86.69 | 0.46 |
|  | 48 |  |  | 87.87 | 1.18 |
|  | 49 |  |  | 88.86 | 0.99 |
|  | 50 |  |  | 89.32 | 0.46 |
|  | 51 |  |  | 91.10 | 1.78 |
|  | 52 |  |  | 91.13 | 0.03 |
|  | 53 |  |  | 90.70 | -0.43 |
| **Shallow Fished** | 1 | 18.92 |  | 4.99 |  |
|  | 2 | 39.25 | 20.33 | 21.56 | 16.57 |
|  | 3 | 47.52 | 8.27 | 29.94 | 8.38 |
|  | 4 | 53.94 | 6.42 | 33.73 | 3.79 |
|  | 5 | 59.69 | 5.75 | 35.04 | 1.31 |
|  | 6 | 64.29 | 4.60 | 36.94 | 1.90 |
|  | 7 | 70.82 | 6.53 | 39.18 | 2.24 |
|  | 8 | 76.88 | 6.06 | 39.83 | 0.65 |
|  | 9 | 81.52 | 4.64 | 42.42 | 2.59 |
|  | 10 | 83.30 | 1.78 | 44.91 | 2.49 |
|  | 11 |  |  | 47.20 | 2.29 |
|  | 12 |  |  | 48.57 | 1.37 |
|  | 13 |  |  | 48.69 | 0.12 |
|  | 14 |  |  | 50.17 | 1.48 |
|  | 15 |  |  | 52.73 | 2.56 |
|  | 16 |  |  | 53.75 | 1.02 |
|  | 17 |  |  | 54.52 | 0.77 |
|  | 18 |  |  | 55.60 | 1.08 |
|  | 19 |  |  | 56.62 | 1.02 |
|  | 20 |  |  | 58.04 | 1.42 |
|  | 21 |  |  | 58.78 | 0.74 |
|  | 22 |  |  | 59.80 | 1.02 |
|  | 23 |  |  | 60.55 | 0.75 |
|  | 24 |  |  | 60.98 | 0.43 |
|  | 25 |  |  | 61.79 | 0.81 |
|  | 26 |  |  | 63.15 | 1.36 |
|  | 27 |  |  | 63.90 | 0.75 |
|  | 28 |  |  | 64.85 | 0.95 |
|  | 29 |  |  | 64.99 | 0.14 |
|  | 30 |  |  | 66.15 | 1.16 |
|  | 31 |  |  | 67.36 | 1.21 |
|  | 32 |  |  | 67.55 | 0.19 |
|  | 33 |  |  | 68.24 | 0.69 |
|  | 34 |  |  | 69.26 | 1.02 |
|  | 35 |  |  | 69.91 | 0.65 |
|  | 36 |  |  | 70.09 | 0.18 |
|  | 37 |  |  | 71.39 | 1.30 |
|  | 38 |  |  | 72.40 | 1.01 |
|  | 39 |  |  | 73.49 | 1.09 |
|  | 40 |  |  | 74.91 | 1.42 |
|  | 41 |  |  | 75.70 | 0.79 |
|  | 42 |  |  | 76.45 | 0.75 |
|  | 43 |  |  | 77.32 | 0.87 |
|  | 44 |  |  | 78.72 | 1.40 |
|  | 45 |  |  | 79.60 | 0.88 |
|  | 46 |  |  | 79.23 | -0.37 |
|  | 47 |  |  | 80.43 | 1.20 |
|  | 48 |  |  | 80.72 | 0.29 |
|  | 49 |  |  | 81.34 | 0.62 |
|  | 50 |  |  | 82.17 | 0.83 |
|  | 51 |  |  | 82.59 | 0.42 |
|  | 52 |  |  | 82.34 | -0.25 |
|  | 53 |  |  | 82.44 | 0.10 |
|  | 54 |  |  | 81.90 | -0.54 |
| **Deep ROA** | 1 | 12.20 |  | 4.94 |  |
|  | 2 | 28.89 | 16.69 | 20.31 | 15.37 |
|  | 3 | 41.08 | 12.19 | 27.91 | 7.60 |
|  | 4 | 48.72 | 7.64 | 31.93 | 4.02 |
|  | 5 | 51.54 | 2.82 | 36.97 | 5.04 |
|  | 6 |  |  | 38.46 | 1.49 |
|  | 7 |  |  | 41.58 | 3.12 |
|  | 8 |  |  | 38.46 | -3.12 |
|  | 9 |  |  | 40.89 | 2.43 |
|  | 10 |  |  | 40.73 | -0.16 |
|  | 11 |  |  | 39.14 | -1.59 |
|  | 12 |  |  | 39.95 | 0.81 |
|  | 13 |  |  | 39.21 | -0.74 |
|  | 14 |  |  | 39.04 | -0.17 |
| **Shallow ROA** | 1 | 18.91 |  | 5.00 |  |
|  | 2 | 42.51 | 23.60 | 18.94 | 13.94 |
|  | 3 | 49.93 | 7.42 | 32.33 | 13.39 |
|  | 4 | 52.76 | 2.83 | 36.71 | 4.38 |
|  | 5 | 54.42 | 1.66 | 39.38 | 2.67 |
|  | 6 |  |  | 39.84 | 0.46 |
|  | 7 |  |  | 42.69 | 2.85 |
|  | 8 |  |  | 44.19 | 1.50 |
|  | 9 |  |  | 45.66 | 1.47 |
|  | 10 |  |  | 45.83 | 0.17 |
|  | 11 |  |  | 48.09 | 2.26 |
|  | 12 |  |  | 47.82 | -0.27 |
|  | 13 |  |  | 48.74 | 0.92 |
|  | 14 |  |  | 49.89 | 1.15 |
|  | 15 |  |  | 52.04 | 2.15 |
|  |  |  |  | 51.46 | -0.58 |
|  |  |  |  | 52.16 | 0.70 |
|  |  |  |  | 53.21 | 1.05 |
|  |  |  |  | 53.15 | -0.06 |
|  |  |  |  | 54.75 | 1.60 |
|  |  |  |  | 56.04 | 1.29 |
|  |  |  |  | 56.78 | 0.74 |
|  |  |  |  | 57.85 | 1.07 |
|  |  |  |  | 58.55 | 0.70 |
|  |  |  |  | 59.80 | 1.25 |
|  |  |  |  | 60.23 | 0.43 |
|  |  |  |  | 61.37 | 1.14 |
